# pHluorin-tagged TRPV1 shines light on capsaicin tachyphylaxis

**DOI:** 10.1080/19336950.2019.1638695

**Published:** 2019-07-08

**Authors:** Francina Agosti, Christophe Altier

**Affiliations:** Department of Physiology and Pharmacology, Inflammation Research Network-Snyder Institute for Chronic Diseases and Alberta Children’s Hospital Research Institute, Cumming School of Medicine, University of Calgary, Calgary, Alberta, Canada

**Keywords:** TRPV1, pHluorin, pain, ion channel trafficking, capsaicin, tachyphylaxis

The Transient Receptor Potential Vanilloid type 1, TRPV1, is a non-selective cation channel sensitive to noxious heat (>42ºC), protons (low pH), lipid metabolites and pungent compounds like capsaicin [1]. TRPV1 is predominantly expressed in specialized primary afferent “nociceptors” neurons whose cell bodies reside within the dorsal root, trigeminal and nodose/jugular vagal ganglia [2]. Activation of TRPV1 by heat or chemical stimuli such as capsaicin mediates burning pain sensation. Moreover, TRPV1 has been comprehensively studied in pathological pain conditions that result from tissue injury or infection [3] and it is well recognized that tissue inflammation enhances both the function and expression of TRPV1 channels in dorsal root ganglion (DRG) neurons, which ultimately amplify pain sensitivity [4]. At the nerve terminal of nociceptors, activation of the channel leads to the release of pro-inflammatory neuropeptides, substance P and CGRP (reviewed in [1]) which have critical immunoregulatory functions, thus highlighting the role of TRPV1 in neurogenic inflammation. In particular, understanding the molecular mechanisms of agonist-induced TRPV1 desensitization or tachyphylaxis is fundamental to gain insight into the mechanism of action of vanilloid analgesics targeting TRPV1.

Many tools have been developed to study TRPV1 sensitization or desensitization in the context of pain and analgesia. Recently, we took advantage of pHluorin, a pH-sensitive ecliptic GFP that fluoresces at neutral pH but dim in acidic cellular compartments to generate a TRPV1 channel whose subcellular distribution can be tracked in real time.

**Figure 1.**
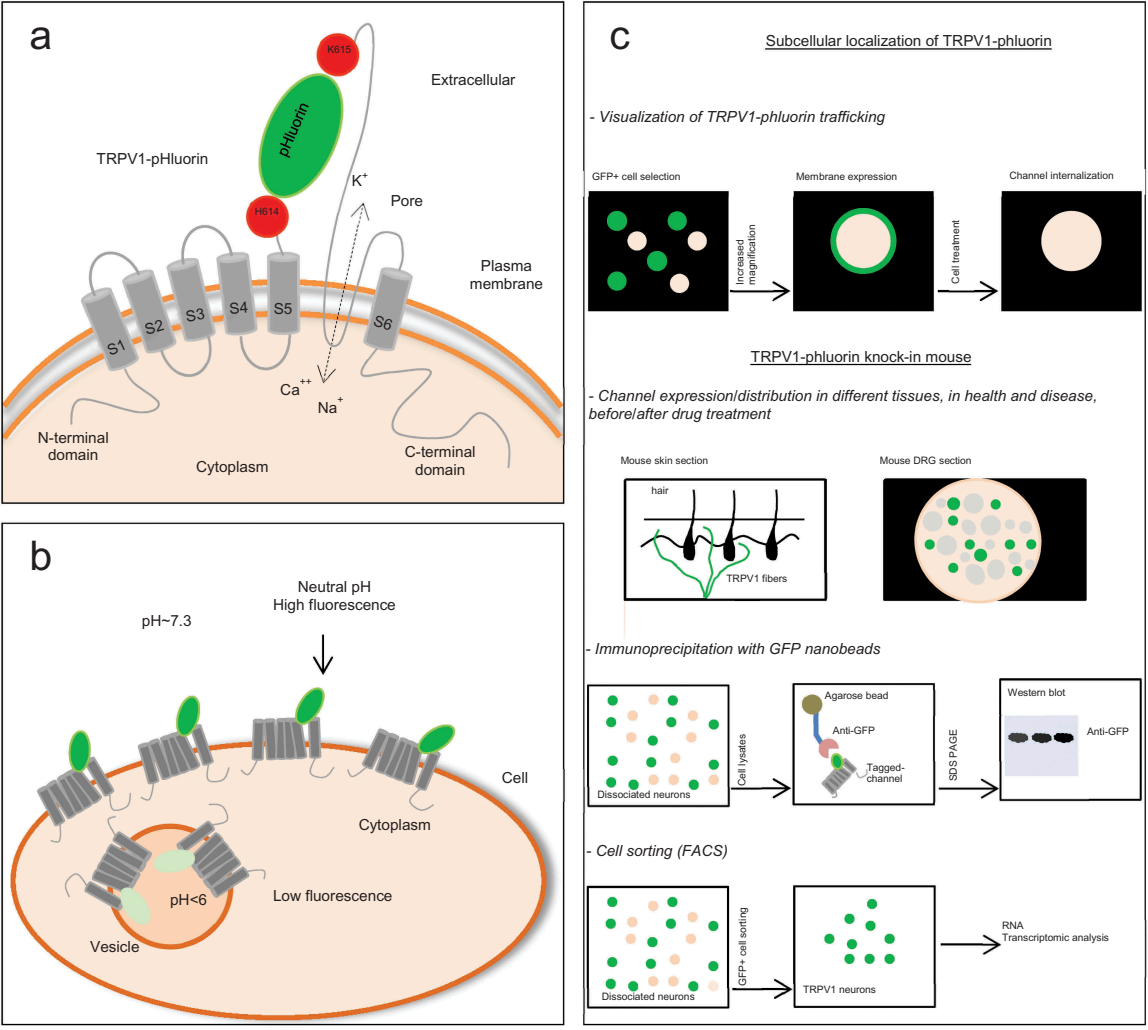
Use and advantage of the TRPV1-pHluorin. (a) Schematic of TRPV1-pHluorinshowing insertion of the pHluorin between the His614 and Lys615 upstream of the pore turret of TRPV1. (b) Intra-organelle pH controls the fluorescence of TRPV1-pHluorin. Acidic pH in the Golgi apparatus, secretory vesicles, and lysosomes decreases the pHluorin fluorescence, whereas neutral pH in the ER and PM increases it. (c) Methodological approaches to examine the trafficking, tissue and cellular distribution of TRPV1 as well as conducting biochemical purification and cell sorting using FACS.

Specifically, our lab reported that the TRPV1-pHluorin construct enables the visualization of surface-expressed TRPV1 channel in transfected HEK cells [5]. We inserted the ecliptic GFP sequence within the extracellular S5-S6 domains, specifically between histidine 614 and lysine 615 residues immediately before the pore turret (Figure 1(a)). The insertion was made by site-directed mutagenesis and strikingly the sensitivity of the channel to capsaicin along with its polymodal mechanism of activation (heat, low pH and ligand) remained unaffected.

In the recent work from Tian and collaborators [6], the authors used electrophysiological assessment of TRPV1 current combined with imaging approaches to examine the activity-dependent regulation of channel function and trafficking at the plasma membrane. Analysis of the dose–response curves for TRPV1 shows that tachyphylaxis mechanisms do not involve a change of channel sensitivity to capsaicin, suggesting that trafficking of the channel might be altered upon tachyphylaxis conditions. To test this hypothesis, the authors took advantage of light-sheet imaging of TRPV1 channels in real time to determine whether the changes in TRPV1 recycling from the plasma membrane reflect the diminution of TRPV1 current in responses to repeated saturated doses of capsaicin.

By creating a TRPV1-pHluorin channel, analogous to the one originally designed by our group, Tian and collaborators showed that TRPV1 recycling at the cell surface was dependent on capsaicin concentration. Low doses (1 µM) of capsaicin allowed rapid insertion of the channel at the cell surface, while high concentrations (10 µM) of capsaicin delayed recovery of its surface expression, thus highlighting the importance of stimulation strength on the trafficking of TRPV1 at the plasma membrane. They next confirmed the stimulation strength dependence of TRPV1 recycling in DRG cultured neurons using voltage clamp recordings.

It was previously described that TRPV1 can interact with synaptotagmin proteins in a Ca2^+^-dependent way [7]. Tian and collaborators studied how different synaptotagmin subtypes contributed to regulating the stimulation strength-dependent kinetics of TRPV1 recycling in transiently expressed HEK cells. They found that during fast recycling induced by low doses of capsaicin, Syt1 facilitated TRPV1 trafficking back to the plasma membrane; while at high doses of capsaicin, Syt7 inhibited TRPV1 recycling to the plasma membrane.

Overall, the work of Tian et al. nicely describes mechanisms underlying the TRPV1 recycling at the plasma membrane upon capsaicin-induced tachyphylaxis and highlights the value of imaging the extracellular pHluorin-tagged TRPV1 to examine channel trafficking. Due to their complex structure, stoichiometry and assembly of subunits, engineering functional ion channels bearing extracellular fluorescent protein has remained challenging, often requiring many unsuccessful attempts. Some examples, however, have allowed us to gain significant insights into the cellular dynamics of these channels in neuronal sub-compartments, including axons and synapses. The groups of Henley and Huganir used a pHluorin-tagged GluA2 as a reporter for AMPA receptor surface expression and endocytosis. In hippocampal neurons, they reported that NMDA receptor activation promotes endocytosis of AMPA receptors, which is an important mechanism underlying long-term synaptic depression [8,9]. Furthermore, the Bourinet lab engineered a Cav3.2-pHluorin mouse line to investigate the tissue distribution and cellular expression of the Cav3.2 channel in both the peripheral and central nervous system (DRG, skin, spinal cord), providing a remarkable tool to validate antibody specificity, to allow electrophysiological recording on isolated and identified Cav3.2-expressing neurons or single cell genetic analysis [10,11].

Our group also highlighted the use of TRPV1-pHluorin as a molecular reporter of TRPV1 expression and trafficking. Developing a TRPV1-pHluorin knock-in mouse could also serve many purposes, such as monitoring TRPV1 expression and trafficking in DRG neurons by visualizing GFP expression or by using GFP targeted nanoparticles. It may also allow cell sorting analysis in pathological settings or facilitate biochemical purification of the channel for proteomic analysis using GFP nano beads (Figure 1(c)).

In summary, ecliptic pHluorin-tagged ion channels provide an innovative and extremely valuable tool for isolating specific subsets of peripheral and central neurons and for monitoring the dynamics of ion channel endocytosis and recycling in living cells.
